# ICD-11 Diagnosis of Body Integrity Dysphoria: A Case Report

**DOI:** 10.1155/crps/6022329

**Published:** 2025-08-27

**Authors:** Jonathan Monk-Cunliffe, Jordan Lin, Anish Patel

**Affiliations:** ^1^Centre for Academic Mental Health, University of Bristol, Bristol, UK; ^2^Bristol Medical School, University of Bristol, Bristol, UK; ^3^Mental Health Liaison Team, North Bristol NHS Trust, Bristol, UK

**Keywords:** body integrity dysphoria, body integrity identity disorder, case report

## Abstract

**Background:** Body integrity dysphoria (BID) is a rare disorder, in which individuals experience a persistent desire to become physically disabled, often through limb amputation. It is now included within the International Classification of Diseases 11th Revision (ICD-11), and this is one of the first case reports to describe the application of these new diagnostic criteria. This also raises the question of treatment pathways for individuals with the disorder, with recognition bringing legitimacy to patients' experience, and responsibility to professionals.

**Case Presentation:** We describe the experience of a 50-year-old man with a long standing desire for his leg to be amputated. He described frustration with the support available, and shame associated with accessing this. After discussions in an online forum he caused dry ice burns to his leg, which resulted in a below-knee amputation. The patient was satisfied with this outcome. All the essential features of ICD-11 diagnostic requirements for BID were met, as well as a number of additional clinical features.

**Conclusions:** New diagnostic criteria appeared both accurate and acceptable to our patient. This formal recognition of the diagnosis presents a new challenge to services about how best to support individuals, and whether there needs to be development of clinical guidance to support clinicians.

## 1. Introduction

Body integrity dysphoria (BID) is a rare disorder, included in ICD-11, which was previously known as body integrity identity disorder. Diagnostic requirements for BID include an intense and persistent desire to become physically disabled in a significant way, with onset by early adolescence accompanied by persistent discomfort, or intense feelings of inappropriateness concerning current non-disabled body configuration, which results in harmful consequences [1].

Our understanding of BID is limited by rarity, shame and moral discomfort about how to respond to individuals with the disorder, but a number of case reports describe a consistent presentation across a range of cultures and settings [2–8]. Individuals report a strong desire to become physically disabled, often through the removal of a physically healthy limb (a number of reports involve the left leg, but other body parts can be involved). When first described in 1977, the condition was considered a paraphilia relating to the desire for amputation, termed ‘apotemnophilia' [9]. However, since then, understanding has changed to recognise that the desire for amputation is a consequence of wanting to restore an individual's body identity [10]. Individuals report that the affected body part does not feel a part of them. The exact prevalence is unclear, but past studies have included hundreds of patients with symptoms, and given the shame associated with the condition, it is likely that many people are not identified [11]. While our understanding of patient experience and treatment options has changed relatively little in the past 20 years [10], there is emerging work to improve our understanding of its aetiology, considering a disruption of the neural networks involved in sensory processing and the representation of the body as a whole [12–14]. Individuals can also show altered pain perception in the affected limb [15].

From existing case reports, it does not appear that psychotherapy or medication significantly changes symptoms [2–11], but the evidence base is limited. While unpalatable to many professionals, there are reports that patients who undergo amputation have a lower perceived disability after amputation, but case numbers are low [16, 17]. There was outrage in the United Kingdom following two amputations of healthy limbs in the 1990s, and while the discussion about whether ‘healthy' limb amputation is morally acceptable has continued, this has not been considered a mainstream treatment option [18–20]. However, a recent case report from Canada describes the elective amputation of two digits with satisfactory outcomes, which may reignite debate on the topic [7, 21]. Some novel treatments are being developed using neurofeedback and augmented reality, but this work is early and small scale [22, 23]. A recent survey of experts on BID suggests that surgical solutions should be considered as part of the treatment pathway, but acknowledges the limited information on how to support individuals with the condition [24]. While this debate goes on patients are left to look elsewhere for support, with online forums filling the gap left by lack of services, and creating an environment where patients may feel driven to self-amputation, which sadly is not uncommon [14].

This case is one of the first descriptions of the application of ICD-11 diagnostic criteria for the disorder, and alongside another recent report from the United Kingdom, shows the utility of these criteria [8]. However, we suggest that the lack of a clear treatment pathway may have contributed to iatrogenic harm for this patient, and are concerned that despite formal recognition of the disorder there remains an absence of guidance for both professionals and patients.

## 2. Case Presentation

A man in his 50s was referred to the liaison psychiatry team following a self-inflicted dry ice burn to his leg, which was subsequently amputated below the knee. He described a longstanding desire for his leg to be amputated and a feeling that his body was more appropriate and complete following amputation, in keeping with the ICD-11 diagnostic criteria for BID.

The patient described thoughts of wanting his left leg to be removed from around the age of 8 or 9, and reported strapping up his leg as a child and standing in front of the mirror. His father had an accident at work before the patient was born, and through childhood the patient witnessed his father living in pain and having multiple operations. The patient felt his father would have been better off if he had an amputation. In early adulthood he had positive social interactions with disabled sports people and activists. He did not feel able to share his experiences of his body with friends and family, but connected online with other people with similar experiences of wanting limbs removed. Over the past decade, he was in regular contact with them through online forums, and watched videos of people with amputations. Following an injury, he used crutches to act as if he was an amputee. He had no other significant past medical history and no relevant family history.

The patient visited his general practitioner (GP), reporting pain in his leg from an old accident at work and requesting an amputation for this reason. The accident did cause the patient ongoing pain, but a major part of his request related to BID, which he did not share at the time. He was seen by the pain team and started on two analgesics, which did not help. He later explained to the GP that he believed he had a diagnosis of BID, but felt like he was not taken seriously. The patient reported he was not referred for psychiatric or psychological support. He found the response of services very difficult, as he experienced a lot of shame and fear when sharing his experiences of his body. He requested elective surgery, but this was not considered an option. Dissatisfied with the support available, he decided to take action so that his leg would be amputated, and took advice on this through online forums. The patient bought dry ice online and used this to cause cold burns to his leg over a number of hours. During the act, he was in discussion with people online, who advised when they felt the leg could no longer be retained by medical treatment, at which point he contacted an ambulance to take him to hospital.

His condition deteriorated, and on admission, the patient was transferred to the intensive care unit (ICU) with a low conscious level and seizures. An MRI scan of his head showed appearances in keeping with small embolic infarcts, which were concluded to be fat emboli due to his injury. After a few days of attempted conservative management, he had a below-knee amputation. He remained in ICU following this, and once his consciousness improved was transferred to a medical ward for rehabilitation.

Once able to discuss the events he reported 'I am now in the body I should be', saying he previously felt he was living in a body he was not meant to be in. The patient described a more complete sense of his body schema, and that he was satisfied with the outcome. He did not describe any sexual arousal from the idea of amputation or amputees. His beliefs are ego-syntonic. He identifies with the gender he was assigned at birth. He did not describe any perceived defects with the limb prior to amputation. There were no signs of cognitive impairment. Throughout multiple reviews by the liaison psychiatry team he did not show signs of a major affective or psychotic disorder. He did not describe any thoughts of suicide, or of self-harm other than the events described above. Differential diagnoses could include depression, psychosis, body dysmorphic disorder, obsessive compulsive disorder or factitious disorder, but as described above and assessed as part of standard liaison psychiatry clinical interviews, his symptoms do not fit with these.

All the essential features of the ICD-11 diagnostic requirements for BID were met, as well as a number of additional clinical features. The patient felt he should have been born with the disability, had simulated being a person with a limb amputation, and had experienced shame and hidden his beliefs from family and friends. Further information on the application of ICD-11 diagnostic requirements to this case are included in Table 1.

At discharge from our service the patient was future focused, thinking about adjustments that could be made to continue his manual job. His care continued with physiotherapy, and he has since participated in an organised charity walk. At 1-year follow-up he said he had no regrets about what happened, but had not expected to have the small vessel infarcts, which he felt had contributed to some short-term memory problems. He experienced some periods of low mood, but related these to family problems unrelated to BID. However, he remained reluctant to discuss his experience with family and friends due to concerns about their reaction. The patient wanted to reflect his frustrations and shame at the limited support available in the community, and the absence of a clear treatment pathway. Once in hospital he was upset by the stigmatising attitudes of some staff, who he felt did not understand his experience.

## 3. Discussion

The patient's symptoms are well described by the new ICD-11 criteria for BID, where someone has an intense and persistent desire to become physically disabled in a significant way, with onset by early adolescence accompanied by persistent discomfort, or intense feelings of inappropriateness concerning current non-disabled body configuration. The diagnosis was well-received, validated the patient's experience and explained this better than other diagnoses. All essential features, and a number of additional features were met, apart from there being a sexual component to the desire (Table 1). Our experience of finding the criteria useful matches expert consensus on the criteria for BID [24].

Despite inclusion in an international diagnostic classification, information on BID remains limited online, with little support available for individuals who believe they may have the condition. In this case the lack of information through established sources meant the patient looked for help in online forums. While it is understandable that his GP may not have been familiar with this rare and recently described condition, the lack of a community treatment pathway also meant that the patient acted on his own. His experiences are similar to those described in other case reports, including another reported in the United Kingdom this year [2, 7, 8, 17]. Evidence on treatment is limited, and it is not possible to know if psychological support or psychotropic medication at the point of presentation would have changed the extent of his symptoms.

There are discussions in the literature about the ethics of elective amputation [7, 18–20], something requested by the patient, and the complexity of this is beyond the scope of this paper. However, if this had been considered, he may not have experienced small vessel infarcts, a consequence of his attempt at self-amputation. Surgical solutions to BID are also considered appropriate in certain circumstances by experts on the condition [24]. It is complicated and challenging to consider how to interpret our duty to do no harm when weighing up ideas around the relief of suffering and iatrogenic harm [14], let alone considering questions of regulation and capacity to consent to such drastic treatment.

However, the inclusion of BID within ICD-11 raises the question of whether there is now an ethical obligation to develop a treatment pathway, acknowledging the limited and low quality evidence on the role of psychotherapy or medication, and giving appropriate scrutiny to the ethical and practical debates about elective amputation, which has been used in another recent case of BID [7].

We recommend that initial work on potential treatment pathways [24] is further developed with involvement from patients, clinicians and professional bodies, to include specific guidance on when and how to consider non-surgical options (including medication and psychotherapy). Further research is needed on the ethics and practicalities of surgical treatment of BID, especially given recent case reports of elective amputation. We also recommend that professional organisations consider the development of patient facing information about BID and how individuals who think they may be affected can access support.

## 4. Strengths and Limitations

A strength of this case report is the repeated assessment of the same patient, and ability to explore his symptoms in depth. The engaged nature of the patient meant that possible diagnosis could be discussed without stigma. However, our assessment occurred following amputation, and information prior to admission to our unit is based on clinical notes. The patient's follow-up continued in a different centre and longer term outcomes are not reported.

## 5. Conclusion

We have described a case applying the ICD-11 criteria for BID, which appeared accurate and acceptable to the patient. This formal recognition of the diagnosis presents a new challenge to services about how best to support individuals who meet criteria, and whether there needs to be development of clinical guidance. There are many challenges when considering potential treatment options, but discussing these is important if we are to provide appropriate care to people with this rare and distressing condition.

## Figures and Tables

**Table 1:** ICD-11 diagnostic requirements.

Category	Criteria	Application to this case
Essential	An intense and persistent desire to become physically disabled in a significant way.	Present
	The desire to be disabled results in harmful consequences through one of:	Self-injury resulting in amputation
	Attempts to actually become disabled through self-injury have resulted in the person putting their health or life in significant jeopardy.	Self-injury resulting in amputation
	Preoccupation with the desire to be disabled results in significant impairment in important areas of functioning.	Relatively good functioning retained, but significant impact on sense of self
	Onset of the persistent desire to be disabled occurs by early adolescence.	Present age 8
	The disturbance is not better accounted for by another mental disorder.	Present
	The symptoms or behaviours are not better accounted for by gender incongruence, by a disease of the nervous system, or by another medical condition.	Present

Additional features	Individuals feel like they should have been born with the desired disability.	Present
	Associated ‘pretending' or simulation behaviour	The patient used crutches to simulate amputation
	Some individuals who attempt to make themselves disabled through self-injury try to cover up the self-inflicted nature of the attempt by making it look like an accident.	The patient was initially reluctant to share their experience with family
	Many individuals with body integrity dysphoria have a sexual component to their desire.	Not present
	Shame about the desire to be disabled is common in individuals with body integrity dysphoria. Some may seek treatment for associated depressive or other symptoms and yet not share their desire to be disabled with their health care provider.	The patient experienced significant shame, and was reluctant to speak with their GP
	Presentation to a health care professional to relieve their distress, to help them actualise their desired disability, or because they have injured themselves in an attempt to become disabled.	Present

## Data Availability

Data sharing is not applicable to this article as no new data were created or analysed in this study.

## Nomenclature

BID: Body integrity dysphoria
GP: General practitioner
ICD-11: International classification of diseases (11th revision)
ICU: Intensive care unit
MRI: Magnetic resonance imaging.
